# Supplementary figures and images for: Pre- and intra -COVID-19 trends of contraceptive use among women who had termination of pregnancy at Charlotte Maxeke Johannesburg Academic Hospital, Johannesburg South Africa (2010–2020)
Source: PLoS One. 2022 Dec 14;17(12):e0277911. doi: 10.1371/journal.pone.0277911 (PMC9750032; doi:10.1371/journal.pone.0277911)

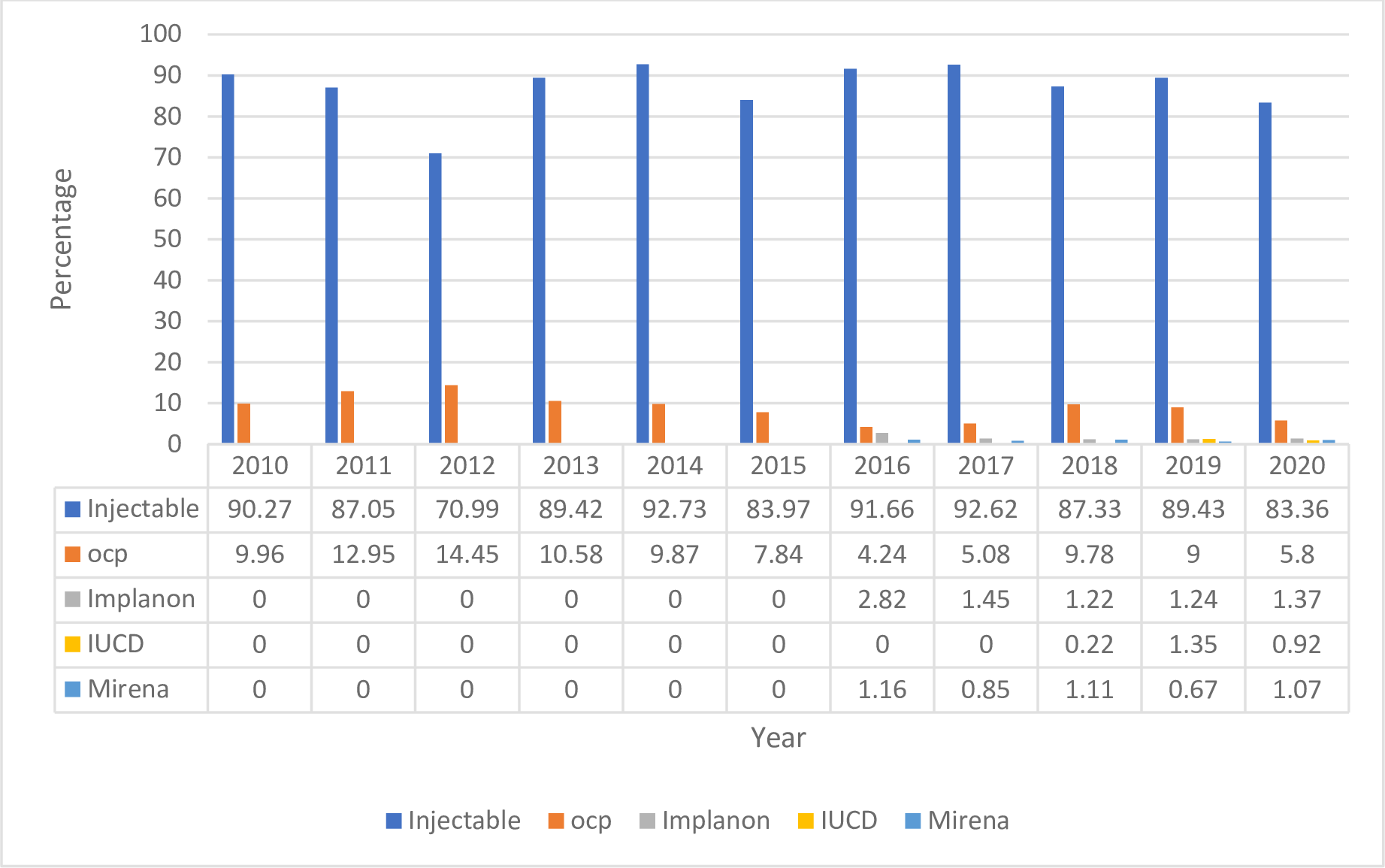

Supplement: S1 Fig — (TIF) [file pone.0277911.s001.tif]
