# Supplementary material for: Pre- and intra -COVID-19 trends of contraceptive use among women who had termination of pregnancy at Charlotte Maxeke Johannesburg Academic Hospital, Johannesburg South Africa (2010–2020)
Source: PLoS One. 2022 Dec 14;17(12):e0277911. doi: 10.1371/journal.pone.0277911 (PMC9750032; doi:10.1371/journal.pone.0277911)
Supplement: S1 Table — (DOCX) [file pone.0277911.s002.docx]

**Supplementary Table 1. Annual trends in number of abortions stratified by age groups and gestational age**

| **Year** | **Number of abortion procedure** | **Age groups (years)** | | | | **Gestational age at procedure (weeks) (annual %)** | |
| --- | --- | --- | --- | --- | --- | --- | --- |
|  |  | **12-18** | **19-25** |  | **>40** | **<13** | **≥13-20** |
| 2010 | 877 | 99 (11.29) | 352(40.14) | 413(47.09) | 25(2.85) | 200(22.81) | 677 (77.19) |
| 2011 | 942 | 109 (11.57) | 340(36.09) | 448(47.56) | 11(1.17) | 199 (20.29) | 782(79.71) |
| 2012 | 929 | 77 (8.29) | 350(37.67) | 470(50.59) | 18 (1.94) | 247 (26.59) | 682(73.41) |
| 2013 | 1452 | 135(9.30) | 556 (38.29) | 761 (52.41) | 86 (5.93) | 333 (22.92) | 1120 (77.08) |
| 2014 | 1441 | 157 (10.89) | 558 (38.72) | 678 (47.05) | 55 (3.82) | 239 (16.47) | 1212 (83.53) |
| 2015 | 1434 | 140 (9.76) | 558 (38.91) | 699 (48.74) | 37 (2.58) | 224 (15.49) | 1222 (84.51) (1446) |
| 2016 | 1278 | 122 (9.55) | 444 (34.74) | 669(54.69) | 38(2.97) | 494 (38.47) | 790 (61.53) |
| 2017 | 999 | 67(6.71) | 405(40.54) | 496(49.65) | 27(2.70) | 999 (100.00) | 0 (0.00) |
| 2018 | 1001 | 79(7.89) | 378(37.76) | 503(50.25) | 32(3.20) | 1001 (100.00) | 0 (0.00) |
| 2019 | 985 | 97(10.05) | 415(42.13) | 452(45.89) | 30(3.05) | 985 (100.00) | 0 (0.00) |
| 2020 | 668 | 75(11.23) | 263(39.37) | 296(44.31) | 27(4.04) | 668 (100.00) | 0 (0.00) |
|  |  |  |  |  |  |  |  |
