# Supplementary material for: Pre- and intra -COVID-19 trends of contraceptive use among women who had termination of pregnancy at Charlotte Maxeke Johannesburg Academic Hospital, Johannesburg South Africa (2010–2020)
Source: PLoS One. 2022 Dec 14;17(12):e0277911. doi: 10.1371/journal.pone.0277911 (PMC9750032; doi:10.1371/journal.pone.0277911)
Supplement: S2 Table — (DOCX) [file pone.0277911.s003.docx]

**Supplementary Table 2. Trends in the contraceptive mix among women who had induced abortion (2010 – 2020)**

|  |  | Oral contraceptive pills | | | Injectable contraceptive | | | Implanon |  |  | Cu IUCD |  | Mirena |  |
| --- | --- | --- | --- | --- | --- | --- | --- | --- | --- | --- | --- | --- | --- | --- |
| Years | Total contraceptive uptake | Number | Proportion  (%) | APC  (%) | Number | Proportion  (%) | APC  (%) | Proportion(%) |  | APC | Proportion(%) | APC | Proportion  (%) | APC |
| 2010 | 853 | 85 | 9.96 | - | 770 | 90.27 | - | 0 |  | - | 0 | - | 0 | - |
| 2011 | 888 | 115 | 12.95 | +30.0 | 773 | 87.05 | -3.57 | 0 |  | - | 0 | - | 0 | - |
| 2012 | 872 | 126 | 14.45 | +11.6 | 619 | 70.99 | -18.45 | 0 |  | - | 0 | - | 0 | - |
| 2013 | 879 | 93 | 10.58 | -26.78 | 786 | 89.42 | +25.96 | 0 |  | - | 0 | - | 0 | - |
| 2014 | 770 | 76 | 9.87 | -6.71 | 714 | 92.73 | +3.70 | 0 |  | - | 0 | - | 0 | - |
| 2015 | 842 | 66 | 7.84 | -20.57 | 707 | 83.97 | -9.45 | 0 |  | - | 0 | - | 0 | - |
| 2016 | 779 | 33 | 4.24 | -45.92 | 714 | 91.66 | +9.16 | 22 |  | - | 0 | - | 9 | - |
| 2017 | 826 | 42 | 5.08 | +19.81 | 765 | 92.62 | +1.05 | 12 |  | -45.45 | 0 | - | 7 | - |
| 2018 | 900 | 88 | 9.78 | +92.52 | 786 | 87.33 | -5.71 | 11 |  | -8.33 | 2 | - | 10 | +42.86 |
| 2019 | 889 | 80 | 9.00 | -7.98 | 795 | 89.43 | +2,40 | 11 |  | 0 | 12 | +500 | 6 | -40.00 |
| 2020 | 655 | 38 | 5.80 | -35.56 | 546 | 83.36 | -6.79 | 9 |  | -18.18 | 6 | -50.0 | 7 | +16.67 |
