# Supplementary material for: Pre- and intra -COVID-19 trends of contraceptive use among women who had termination of pregnancy at Charlotte Maxeke Johannesburg Academic Hospital, Johannesburg South Africa (2010–2020)
Source: PLoS One. 2022 Dec 14;17(12):e0277911. doi: 10.1371/journal.pone.0277911 (PMC9750032; doi:10.1371/journal.pone.0277911)
Supplement: S3 Table — (DOCX) [file pone.0277911.s004.docx]

**Supplementary Table 3: Actual and predicted values of abortion using linear regression modelling**

| Year | Actual Number of abortion procedures | Predicted number of abortions procedures | Contraceptive prevalence | Predicted contraceptive prevalence |
| --- | --- | --- | --- | --- |
| 2014 | 1441 | 1465.2 | 53.4 | 51.3 |
| 2015 | 1434 | 1355.0 | 58.7 | 59.8 |
| 2016 | 1278 | 1244.8 | 61.0 | 68.4 |
| 2017 | 999 | 1134.6 | 82.7 | 76.9 |
| 2018 | 1001 | 1024.3 | 89.9 | 85.5 |
| 2019 | 985 | 914.1 | 90.3 | 94.0 |
| 2020 | 668 | 803.9 | 98.1 | 102.6 |
