# Supplementary material for: Pre- and intra -COVID-19 trends of contraceptive use among women who had termination of pregnancy at Charlotte Maxeke Johannesburg Academic Hospital, Johannesburg South Africa (2010–2020)
Source: PLoS One. 2022 Dec 14;17(12):e0277911. doi: 10.1371/journal.pone.0277911 (PMC9750032; doi:10.1371/journal.pone.0277911)
Supplement: Attachment — Submitted filename: rebuttal letter plos one.docx [file pone.0277911.s005.docx]

Department of Obstetrics and Gynaecology

University of Witwatersrand

Johannesburg, South Africa

17^th^ September, 2022.

The Editor in Chief,

PLOS ONE Journal

**REBUTTAL LETTER**

We are very grateful to the Editor and the two reviewers for their comments on our manuscript. Below is our response to the comments raised by the academic registrar and the two reviewers. We have tried to address all the comments raised and believe that our paper has really improved considerably. We would also be happy to make further corrections to our paper if necessary.

We hope our response would be granted the needed attention and consideration it deserves.

Yours Sincerely,

On behalf of all the authors

Dr Baffour-Duah.

**ACADEMIC EDITOR:**

**A # Abstract**

A. **Methods**
1. **Line 25, there is a punctuation error and modified like this, annual contraceptive uptake rate(stratified by types)**

The sentence was modified and the punctuation error addressed. We are very grateful.

2. **Line 28 also you missed punctuation, correct it.**

We have corrected the punctuation omission. Thank you very much

B. **Results**
1. **In line 30, the number is not clear to the reader. Did you say that 49.26%?**

We have clarified the statement. Yes we meant 49.26%. Thank you

2. **Lines 35-36, you make…. annum from 53.45 per a hundred clients in 2014 to 98 per 100 hundred clients. You can change from ….53.45 per 100 clients in 2014 to 98 per 100….**

We have effected the changes. Thank you.

**What is your cut point to say the majority……. you should rewrite again**

We have addressed the statement and replaced the majority with most.

C. **Introduction**
1. **Your introduction is too long, shorten it**

We have worked on the introduction. We have tried to focus on the key issues related to the content of the manuscript. We are very grateful.

2. **Line 50 & 60, there is a grammar error, contraception is a very important prevention strategy for maternal mortality…..**

We have edited that statement. Thank you

3. **Line 54 too late information, we are in 2022, so, you included the updated information**

We have removed the entire statement to also help in reducing the content of the introduction. Thank you.

4. **Line 57-58, you should correct it like this. It is expected that the liberalization of access to induced abortion services can reduce unsafe abortion and its complications.**

We have addressed the comment and effected the change.

5. **In line 61, spelling error, line 62, punctuation error, line 65-68, need a reference, line, 78, you said that the prevalence rate, you should use either one. Prevalence is quite different to rate, and in line 100 punctuation**

We have addressed all the punctuation errors, referenced lines 65-68 and remove the rate from the sentence.

D. **Methods**
1. **Line 114, is the province city a capital city? Revise it. Line 121-122 grammatically isn’t correct.**

Johannesburg is not the capital city of South Africa. It is a provincial capital of Gauteng and the largest city in south Africa. We have addressed this and the grammatical errors as well. Thank you.

2. **Line 145, do have waivers for identified cases?**

We did get approval from the hospital CEO and from WITS university HREC. We only analysed the records available . confidentiality of the data was ensured as we didn’t use names or other identifiers. The study was a retrospective cross-sectional study. We reviewed the nursing records and summaries of the induced abortion cases that were performed for the past eleven years from 1 January 2010 to 31 December 2020. The annual number of patients who presented for termination of pregnancy, annual number of patients who had contraception afterwards and the various methods that were accepted were extracted into a spreadsheet for analysis. Confidentiality of the data was ensured as names and other identifier were not utilized. The data obtained was aggregated and fully anonymized. The ethics committee waved the requirements for informed consent from the clients to have the data from the nursing records used in the research.

E. **Results**
1. **Line 208 grammatically inst correct. 219-226, needs modification, first you should write test, then followed by a table and figure. You should address all tables and figures based on the above comments.**

we corrected the grammatical error, amended the text and table arrangements to comply with the style requirements.

F. **Discussion**
1. **Line 460, is number (226) or percent**

The 226 is a number not a percent. Thank you.

**REVIEWER #1**

**Title**

**The short title seems better, it is more concise**

We are very grateful.

**Abstract**

**Background**

**Lines 16-17:” the pattern of contraceptive uptake can guide interventions among these group of vulnerable individual”. the authors use “these group” without prior mention of any specific group**

We have amended the statement and removed these group.

**Methods**

**Use uppercase for COVID-19. write it in full at first use.**

We have addressed this in the manuscript. Thank you.

**Results**

**There is no report on association in the results.**

We noted this and have corrected it in manuscript. Thank you.

**Introduction**

**Lines 70-72:” Furthermore, the 1996 constitution of the republic of South Africa, Act 108 of 1996, promotes reproductive rights and the rights of access to reproductive health care.” is this ACT available online? If yes, could you include a reference for readers interested in obtaining further information about this ACT?**

Most of the ACTS in the South African 1996 constitution are available online including ACT 108. We have included a reference in this context with regards to the promotion of reproductive rights and the rights of access of reproductive health care. Thank you for drawing our attention to this.

**Lines 74,76,90-91: some grammatical errors noted: government supplies; the contraceptive prevalence rate …remains; use of although and however in same sentence.**

We thank you for drawing our attention to them. We have made the necessary corrections.

**Methods:**

**The authors should consider moving the ethical considerations section to the end of the methods section**

The ethical consideration section follows the study procedure which ends the method section. Thank you.

**Line 161: typo noted: joint**

We have corrected it to Join. Thank you

**Statistical analysis**

**Two methods were used to predict the expected numbers for 2020. what is the rationale for using both? What advantages do the methods offer, over other prediction models?**

Indeed there are many prediction modelling that have been described in the literature. However, join point regression was utilised as a novel tool for predicting trends. We then utilised the linear regression models to compare results and we still had similar outcomes. Thank you

**Did the trend analysis account for changes in underlying at-risk population? It is highly likely that the population in catchment area may varied from year to year.**

we thank the reviewer for this comment. Our analysis was on trends in contraceptive uptake rates among abortion cases. Since we utilised rates per abortion cases and not the actual numbers of contraceptive uptake, we believe that the trends analysis had corrected for the at risk population who were the clients that came for abortion services. Thus changes in the abortion clients who were the at risk population will also reflect in our trends.

**Results** :

**Line 270: grammatical error noted; the uptake started increasing remained high until 2015….. table 3 column 4 row 3: error is reported in figure. There is no figure 4**

Grammatical errors noted, corrections made. Figure 4 has been corrected and the error in table 3 rectified now. Thank you.

**Discussion:**

**Line 419: grammatical error noted; contraceptive uptake has increased to about 98%…… Line 514: grammatical error noted; annual number of induced abortions**

The corrections have been made. Thank You

**Reviewer #2**

**Overall , the manuscript addresses a very important subject in the area of reproductive health**

We are very grateful.

**The research questions and the findings are well presented by the authors**

We are very grateful.

1. **the authors should clarify whether the study was able to determine the types of contraceptives available at the study site. If no, data is available to support this claim, then they cannot recommend that “other long acting contraceptives should be made available” when they have not ascertain the currently available options.**

We have amended our statement since we don’t have data to support the types of contraceptive availability at the study site. Thank you

2. **The authors should do a thorough proof reading of the manuscript and ensure consistency in the use of terminologies eg covid -19**

We did as you suggested. Thank you.
