# Supplementary material for: Pre- and intra -COVID-19 trends of contraceptive use among women who had termination of pregnancy at Charlotte Maxeke Johannesburg Academic Hospital, Johannesburg South Africa (2010–2020)
Source: PLoS One. 2022 Dec 14;17(12):e0277911. doi: 10.1371/journal.pone.0277911 (PMC9750032; doi:10.1371/journal.pone.0277911)
Supplement: Attachment — Submitted filename: Rebuttal letter.docx [file pone.0277911.s006.docx]

Department of Obstetrics and Gynaecology

University of Witwatersrand

Johannesburg, South Africa

31^st^ October, 2022.

The Editor in Chief,

PLOS ONE Journal

**REBUTTAL LETTER**

We are very grateful to the Editor and the reviewers for their comments on our manuscript. Below is our response to the comment raised by the academic registrar and the reviewers. We have tried to address the comments raised and believe that our paper has really improved considerably.

We hope our response would be granted the needed attention and consideration it deserves.

Yours Sincerely,

On behalf of all the authors

Dr Baffour-Duah.

**Reviewer #1**

**Lines 94-97**

**‘ ALTHOUGH, virtually all contraceptive technologies can be safely utilized by clients as post abortion contraceptives, BUT the pattern of contraceptive uptake may be influenced by health system system concerns such as distribution and availability of the various contraceptive types, and the individual experiences and preference’**

**I suggest that you delete ‘ALTHOUGH’ or ‘BUT’**

We have changed the sentence. Thank you.
